# Respiratory Disparity? Obese People May Not Benefit from Improved Air Quality

**DOI:** 10.1289/ehp.121-a283

**Published:** 2013-09-01

**Authors:** Carol Potera

**Affiliations:** **Carol Potera**, based in Montana, has written for *EHP* since 1996. She also writes for *Microbe*, *Genetic Engineering News*, and the *American Journal of Nursing*.

About a third of U.S. adults and 17% of U.S. children were obese in 2009–2010, according to the Centers for Disease Control and Prevention.^1^ Obesity is associated with reduced lung function^2^ and other health conditions, including asthma^3^^,^^4^ and cardiovascular disease.^5^ Exposure to ambient particulate matter is associated with similar health effects, which may be exacerbated by obesity.^6^^,^^7^

While promoting various types of negative health effects, the effects of obesity also may mask those of beneficial interventions. In this issue of *EHP*, Swiss researchers report that when air quality improves, so does lung function in adults, but only in people with a low or normal body mass index (BMI).^8^

The team used data from the Swiss Study on Air Pollution and Lung Disease in Adults (SAPALDIA), a population-based study launched in 1991. The ongoing longitudinal study tracks the respiratory health of several thousand adults who live in eight geographic regions of Switzerland.^8^

The latest analysis of the SAPALDIA cohort compares spirometry data collected in 1991 and 2002 for 4,664 participants. Lung-function tests included forced expiratory volume in 1 second (FEV_1_),^9^ forced vital capacity (FVC),^10^ and average forced expiratory flow over the middle half of the FVC (FEF_25–75_).^11^

The investigators estimated changes in each participant’s average exposure to coarse particulate matter (PM_10_) outdoors at home between 1991 and 2002 using air quality data and dispersion models developed by the Swiss government. The median PM_10_ concentration was 5.3 µg/m^3^ lower in 2002 than in 1991. Air quality improved more in cities than in the alpine regions of Davos and Montana, which had cleaner air to start with.^12^ Participants’ BMIs were used to assess obesity, with most people gaining weight during the 10-year period.^8^

**Figure 1:**
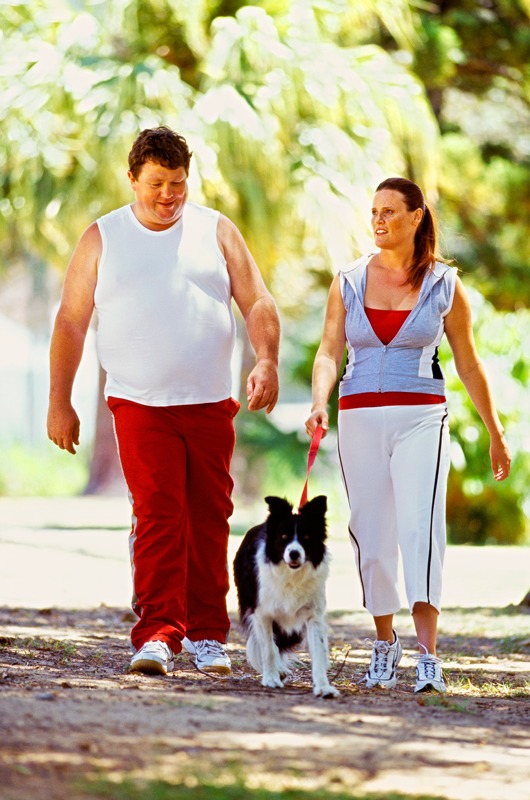
Improved air quality may not be enough to compensate for lung function reduced as a result of excess weight. © Corbis

Lung function generally declines with age. In the SAPALDIA cohort this natural age-related decline was slowed in people breathing cleaner air, but only in those with a BMI of less than 24 (i.e., those who were normal- or underweight). Yearly changes in some lung-function parameters, particularly those related to the small airways (such as FEF_25–75_), were slowed by up to 30% in people with low or normal BMIs. Overweight and obese people showed no benefit to lung function from breathing cleaner air, meaning their annual age-related decline in lung function was not slowed down.^8^

The results “suggest that attenuation of age-related lung function decline due to improved air quality may be observable only in normal-weight and underweight persons,” conclude the authors.^8^ The connection needs to be confirmed with more studies that use direct measures of obesity rather than participants’ report of their own weight, says study leader Tamara Schikowski, a research scientist at the Swiss Tropical and Public Health Institute in Basel.

Excess weight is associated with reduced ability of the lung to stretch, which increases the mechanical work needed to breathe.^13^ Improved air quality may not be enough to compensate for these physical changes in overweight and obese people. Additionally, excess weight^14^ and air pollution^15^ are both associated with chronic inflammation and together may be more likely lead to permanent damage of lung tissue, reducing the benefits of breathing cleaner air, Schikowski suggests.

“The strength of the Swiss study is that the population is large, and standardized spirometric and air pollution data are available,” says Norbert Berend, an emeritus professor and director of Respiratory Research at the George Institute of Global Health, University of Sydney, Australia. The authors’ speculation that systemic inflammation due to obesity may prevent the beneficial effects of reduced pollution “has important implications and needs to be followed up with further studies,” Berend notes.
